# Hypoxia-mediated mitochondria apoptosis inhibition induces temozolomide treatment resistance through miR-26a/Bad/Bax axis

**DOI:** 10.1038/s41419-018-1176-7

**Published:** 2018-11-13

**Authors:** Xin Ge, Min-Hong Pan, Lin Wang, Wei Li, Chengfei Jiang, Jun He, Khaled Abouzid, Ling-Zhi Liu, Zhumei Shi, Bing-Hua Jiang

**Affiliations:** 1 Key Laboratory of Human Functional Genomics of Jiangsu Province, and Department of Pathology, Nanjing Medical University, Nanjing, Jiangsu China; 2 Department of Pathology, Carver College of Medicine, The University of Iowa, Iowa, IA 52242 USA; 3 Department of Pathology, The First Affiliated Hospital of Nanjing Medical University, Nanjing, China; 4 Academy of Medical Sciences, Zhengzhou University, Zhengzhou, China; 5 Department of Pathology, Affiliated Drum Tower Hospital of Nanjing University Medical School, Nanjing, China; 6 Department of Pathology, Anatomy and Cell Biology, Thomas Jefferson University, Philadelphia, PA USA; 7 Pharmaceutical Chemistry Department, Faculty of Pharmacy, Ain Shams University, Cairo, Egypt; 8 Department of Neurosurgery, The First Affiliated Hospital of Nanjing Medical University, Nanjing, China

## Abstract

Glioblastoma multiforme (GBM) is one of the most hypoxic tumors of the central nervous system. Although temozolomide (TMZ) is an effective clinical agent in the GBM therapy, the hypoxic microenvironment remains a major barrier in glioma chemotherapy resistance, and the underlying mechanisms are poorly understood. Here, we find hypoxia can induce the protective response to mitochondrion via HIF-1α-mediated miR-26a upregulation which is associated with TMZ resistance in vitro and in vivo. Further, we demonstrated that HIF-1α/miR-26a axis strengthened the acquisition of TMZ resistance through prevention of Bax and Bad in mitochondria dysfunction in GBM. In addition, miR-26a expression levels negatively correlate with Bax, Bad levels, and GBM progression; but highly correlate with HIF-1α levels in clinical cancer tissues. These findings provide a new link in the mechanistic understanding of TMZ resistance under glioma hypoxia microenvironment, and consequently HIF-1α/miR-26a/Bax/Bad signaling pathway as a promising adjuvant therapy for GBM with TMZ.

## INTRODUCTION

Glioblastoma multiform (GBM), the most malignant form of primary brain tumor in adults, is highly aggressive and currently incurable. Although notable advancements have been developed for GBM in the past 30 years, the median survival of 12–15 months has not been appreciably improved^1^. The chemo-resistance is still the worst barrier in GBM treatment. Temozolomide (TMZ), the current first-line chemotherapeutic agent for GBM, is a DNA alkylating antineoplastic drug that induces DNA strand breaks during cell replication and promotes cell apoptosis^2,3^. The crucial factors of TMZ resistance are comprised of weak drug penetration due to hypoxia inside the tumor and tumor cells’ strongly anti-apoptosis activity. Previous study indicated chemo-resistance can be potentiated by hypoxia, a common feature in solid tumor. The hypoxia-inducible factors (HIFs), the key transcriptional regulator in response to hypoxia, facilitate tumor progression and associate with poor survival^4^. The suppression of HIF-1α has been investigated to sensitize GBM cells to TMZ treatment^5^. However, the underlying mechanism still remains elusive. Thus, the understanding of the association between hypoxia and TMZ resistance is essential to improve current anticancer strategies in GBM.

To survive in hypoxic conditions, cancer cells often avoid apoptosis by altering their intrinsic gene expression patterns. Recent studies shown that hypoxia-induced the microRNAs (miRNAs) expression and these hypoxia-regulated miRNAs (HRMs) may be responsible for the modulation of tumor-related genes in a low-oxygen environment in GBM^6,7^. MiRNAs, the 18–22nt small non-coding RNAs for regulating the development of multiple tumors, are known as post-transcriptional modulators by inhibiting translation of target mRNAs^8–11^. The aberrant expression of hypoxia-regulated miRNAs play key roles in GBM development, including cell proliferation, apoptosis, and invasion^12,13^ as well as the sensitize to TMZ in GBM therapy^14–16^. Notably, miR-26a was identified to be strongly correlated with malignancy in human GBM and received much attention in recent years by targeting PTEN^17^. Our previous study also demonstrated that miR-26a promoted tumor growth and angiogenesis in glioma^18^. However, the mechanism of miR-26a responses to hypoxia in GBM cells, and the effects of miR-26a to TMZ treatment have never been determined.

Apoptosis resistance is an important characteristic of tumor cells. Mitochondria apoptosis is regulated by Bcl-2 family proteins which control the release of cytochrome (Cyt) *c* from mitochondria. Bax and Bad are known to mediate intrinsic mitochondrion-dependent apoptosis^19,20^. They will permeabilize the outer membrane and trigger the release of cytochrome *c* and subsequently cascade activation of caspase family, which leads to activation of key downstream proteins and consequent genomic DNA damage^19,21^. Recent studies have shown that treatment with TMZ may change the mitochondrial pathway of apoptosis by Bax and Bad^22^. Nonetheless, the specific mechanism of Bax and Bad regulation is still unexplored.

In the present work, we sought to investigate the relationship between hypoxia and GBM chemotherapy resistance, we plan to investigate whether miR-26a upregulation in hypoxic microenvironment could promote the TMZ resistance in GBM cells and whether it may protect mitochondria dysfunction by inhibiting pro-apoptosis factors such as Bax and Bad. Our findings would provide insights into GBM chemo-resistance and clinical implication for cancer therapy in the future.

## RESULTS

### Hypoxia induces resistance of glioma cells during temozolomide treatment

**Fig. 1:**
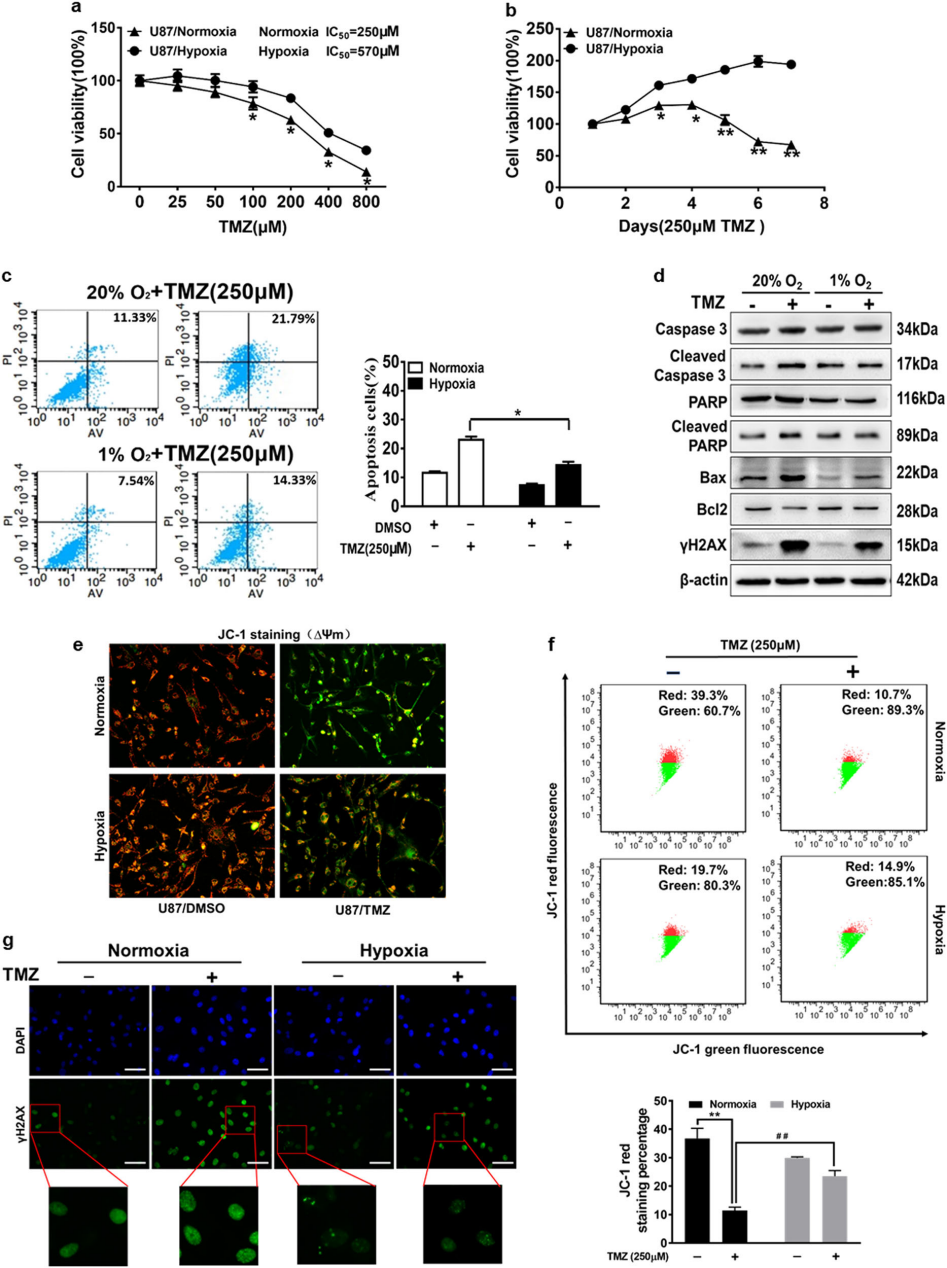
Hypoxic microenvironment improves tolerance of glioma cells to temozolomide though decreased DNA damage and protection mitochondria function. **a** U87MG cells exposed to normoxic (20% O_2_) or hypoxic (1% O_2_) conditions were pretreated with TMZ of various concentration for 72 h and subjected to CCK8 Assay. **b** U87MG cells exposed to normoxic or hypoxic condition were pretreated with TMZ (250 μM) for indicated time points, then subjected to CCK8 Assay. **c** The apoptotic rates of U87MG cells exposed to normoxic or hypoxic condition upon treatment with DMSO or TMZ (250 μM) for 72 h were measured by flow cytometry. **d** Western blotting analysis showing levels of caspase-3, cleaved caspase-3, PARP, cleaved PARP, Bax, Bcl-2, and γH2AX after indicated treatment. **e**, **f** The cells with treatment as indicated were stained with JC-1 probe and detected by fluorescence microscope and flow cytometer. Red fluorescence represents the aggregate form of JC-1, indicating high mitochondrial membrane potential (ΔΨm). Green fluorescence represents the monomeric form of JC-1, indicating impaired mitochondrial membrane potential (ΔΨm). **g** Immunofluorescence staining of γH2AX in U87MG cells exposed to normoxia or hypoxia and TMZ (250 µM) or not for 72 h (magnification, ×400). Scale bar = 100 μm γH2AX: Green; DAPI: Blue. Data were presented by means ± SEM. in triple experiments. Asterisk indicated significant difference at *P* < 0.05 between normoxia group and hypoxia group. Double asterisk indicated significant difference at *P* < 0.01 between control group and TMZ treatment group in normoxia. Double hash indicated significant difference at *P* < 0.01 between normoxia group and hypoxia group treated with TMZ

The exposure of U87MG cells to hypoxia (1% O_2_) resulted in a marked change of cell viability compared to normoxia (20% O_2_) cultured cells. To evaluate the effects of hypoxia on glioma chemo-resistance, we found significantly increased levels of HIF-1α from 6 to 48 h post hypoxia treatment (Fig. S1a)^23^ and hypoxia decreased glioma cells sensitivity to different doses of TMZ (Fig. 1a). Subsequently, cell growth rate in the presence of TMZ (250 μM) was assayed at different time points, and the results indicated that hypoxia-induced glioma cell survival upon TMZ treatment (Fig. 1b). The colony formation and EdU proliferation assay also illustrated that U87MG cells exposed to TMZ in hypoxic condition increased the proliferating ability compared with that of normoxic condition (Figs S1b-c). In order to test whether the resistance to TMZ under hypoxia is caused by cell apoptosis, FACS analysis showed that the apoptotic rates of U87MG cells exposed to TMZ under the normoxic condition were higher than that under the hypoxic condition (Fig. 1c). To determine the mechanism of TMZ-induced apoptosis under hypoxia, we found that the exposure of U87MG cells to TMZ treatment increased cellular cleaved caspase-3, cleaved PARP, and Bax expression levels, but decreased Bcl-2 expression levels in normoxic condition. However, hypoxia attenuated TMZ treatment-induced cell apoptosis (Fig. 1d). Bcl-2 protein family can protect cells from apoptosis via outer mitochondrial membrane permeabilization^24^. Thus, we detected mitochondrial membrane potential (ΔΨm) changes in TMZ-treated cells using fluorescence microscope and flow cytometer after JC-1 staining, since JC-1 was able to selectively enter mitochondria and reversibly shift the color between red and green as the membrane potentials alter from high to low. The mitochondrial membrane potentials are indicated by the red/green fluorescence intensity ratio. Compared with normoxic condition, cells treated with TMZ under the hypoxia condition showed a less impairment of ΔΨm with 50% higher levels of red fluorescence, which indicates the cell survival (Fig. 1e, f). These results suggest that hypoxia protects mitochondrial function and decrease the TMZ-induced apoptosis.

TMZ is a kind of alkylating agent to cause DNA damage and cell apoptosis. The phosphorylation of histone family member X at serine 139 (γH2AX) is widely used as a molecular marker for DNA double strands break (DSB)^25^. Interestingly, our results showed that TMZ treatment induced the expression levels of γH2AX, while hypoxia reduced the augment of γH2AX in TMZ treatment U87MG cells relative to the control cells (Fig. 1d). Furthermore, Immunofluorescence staining of γH2AX foci showed that the number of γH2AX foci was increased in the TMZ-treated U87MG cells and was attenuated under hypoxic condition compared with normoxic condition, suggesting that hypoxia helped inhibition of DNA damage in U87MG cells (Fig. 1g). Thus, our data indicate that hypoxic microenvironment protects the glioma cells from apoptosis to TMZ treatment though protecting mitochondria function and decreasing apoptosis and DNA damage.

### Mechanism of miR-26a induction by hypoxia

**Fig. 2:**
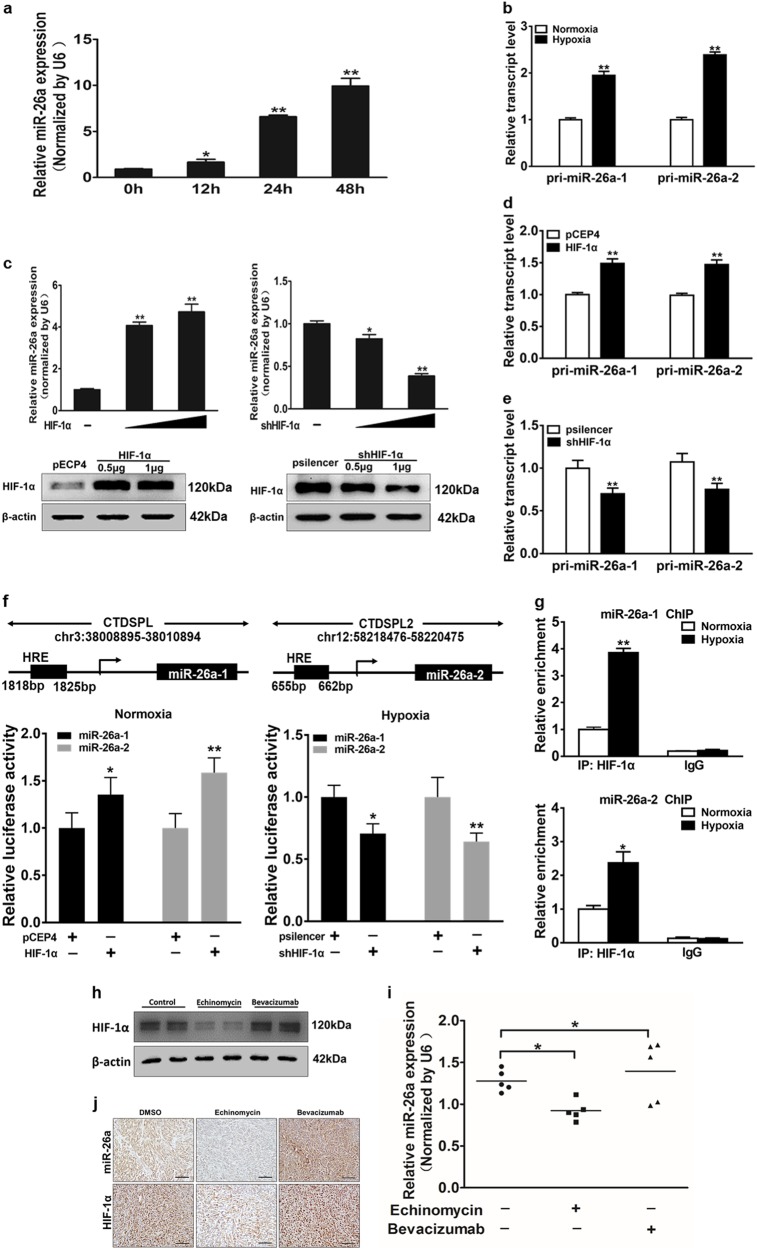
MiR-26a expression is upregulated under hypoxia through HIF-1α in glioma cells. **a** Quantitative real-time PCR (qRT-PCR) was performed to measure time-dependent expressions of miR-26a after exposure to hypoxia for 0, 12, 24, or 48 h. **b** Measurement of pri-miR-26a-1 and pri-miR-26a-2 among U87MG cells were showed in cells exposed to hypoxia compared with normoxia. **c** Upper: qRT-PCR was used to detect the expression of miR-26a after transfection. Lower: The HIF-1α protein levels were examined by western blotting to confirm the plasmids transfection efficiency. **d**, **e** Expression levels of pri-miR-26a-1 and pri-miR-26a-2 were measured in cells overexpression or interference of HIF-1α. **f** U87MG cells were respectively transfected with miR-26-1/2 promoter reporter plasmids and pCEP4-HIF-1α or shHIF-1α (psilencer), and relative luciferase activities were determined. **g** Cells were exposed to normoxia or hypoxia for 24 h. ChIP assay was performed using antibodies against IgG or HIF-1α followed by analysis of qRT-PCR. **h** Nude mice were inoculated with U87MG cells. And at day 14th after implantation, nude mice were treated with echinomycin (0.12 mg/kg) or bevacizumab (10 mg/kg) every 2 days for 16 days. HIF-1α expression was tested by western blotting to confirm the establishment of the animal models. **i** Expression levels of miR-26a in tumor masses were measured by qRT-PCR. **j** HIF-1α and miR-26a levels and locations in tumor tissues were measured by in situ hybridization and immunohistochemistry, respectively. Scale bar = 100 μm. Data were presented by means ± SEM. in triple experiments. Asterisk or hash indicated significant difference at *P* < 0.05 compared with DMSO group. Double asterisk indicated significant difference at *P* < 0.01 compared with control group

In order to test the differential expression of different miRNAs in glioma chemo-resistance under hypoxic conditions, quantitative real-time PCR (qRT-PCR) was used to detect known key miRNAs in glioma. We found miR-26a was one of the most significantly changed miRNAs, which is closely associated with glioma clinical stages, thus we selected miR-26a for further analysis (Fig. S2a). We found that miR-26a was dramatically up-regulated after 12 h or longer time under the hypoxia condition, but was not changed significantly up to 6 h under the normoxia condition (Fig. 2a and S2b). Analysis of genome data indicated that the mature form of miR-26a is generated from 2 separate loci, miR-26a-1 and miR-26a-2, co-expressed with their host genes CTDSPL and CTDSP2, correspondingly. Thus, we detected the primary forms and host genes of miR-26a under hypoxia and found the expression levels of pri-miR-26a-1, pri-miR-26a-2, CTDSPL, and CTDSP2 were increased as the same trend with miR-26a (Fig. 2b and S2c). HIFs are known to regulate a large expression profile of genes under hypoxia^26^. To investigate whether HIF-1 is involved, we transfected the cells by overexpressing or depressing HIF-1α expression under normoxia or hypoxia. Western blotting showed plasmids transfection efficiency. Overexpression of HIF-1α increased miR-26a expression, and HIF-1α shRNA decreased miR-26a levels (Fig. 2c). The expression of pri-miR-26a-1, pri-miR-26a-2, CTDSPL, and CTDSP2 were up-regulated during overexpression of HIF-1α, whereas HIF-1α shRNA had the opposite effect (Fig. 2d, e and S2d, e). Furthermore, we found that there are several HIF-1α response elements (HREs) in the promoter region of miR-26a-1 and miR-26a-2 using bioinformatics analysis. We selected regions containing potential HRE sequences of miR-26a-1 and miR-26a-2 promoter and cloned into a luciferase reporter vector. The luciferase activities of miR-26a promoter were increased by HIF-1α overexpression under normoxia condition but were decreased by shHIF-1α even under hypoxic condition (Fig. 2f). ChIP assay was performed using antibodies against IgG or HIF-1α followed by analysis of qRT-PCR and showed that HIF-1α directly bound to the promoter region of miR-26a and enhanced the expression of miR-26a (Fig. 2g). To explore whether HIF-1α is involved in mediating miR-26a expression in vivo, nude mice were inoculated with U87MG cells, after two weeks, treated with echinomycin (0.12 mg/kg) or bevacizumab (10 mg/kg) every 2 days for 16 days. Then western blotting and qRT-PCR were conducted, indicated that protein levels of HIF-1α and mRNA expression of miR-26a in tumors were repressed by echinomycin treatment and increased by bevacizumab treatment (Fig. 2h, i). The expression levels of HIF-1α and miR-26a in tumor tissues were analyzed by immunohistochemistry and in situ hybridization, respectively, which were consistent with previous results (Fig. 2j). These results indicate that HIF-1α also induces miR-26a expression in vivo. Taken together, the results demonstrate that HIF-1α is a key upstream inducer of miR-26a expression in glioma.

### MiR-26a is important for TMZ treatment resistance

**Fig. 3:**
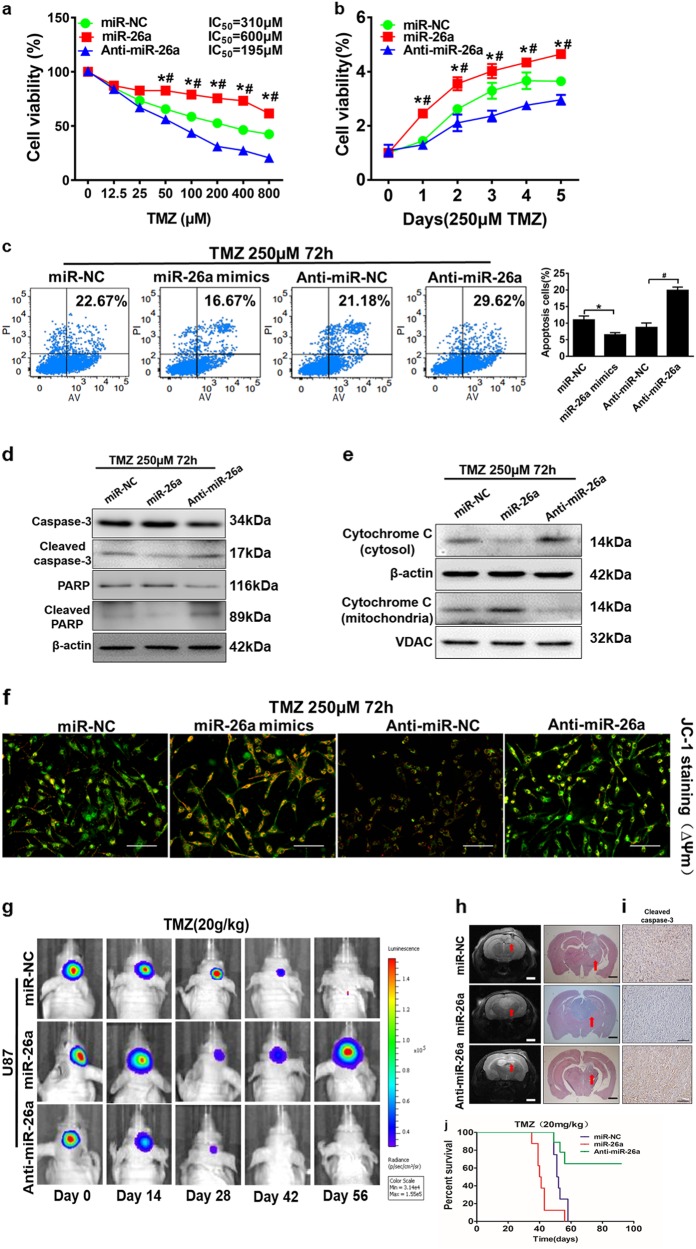
MiR-26a protects glioma cells mitochondrial function and promotes TMZ resistance in vitro and in vivo. **a**) CCK8 assay was performed in U87MG cells stably expressing miR-NC, miR-26a, or anti-miR-26a inhibitor, with TMZ treatments at different concentrations for 72 h. **b** The cell proliferation in 250 µM TMZ treatments was tested every 24 h in cells respectively. **c** U87MG cells were transfected with miR-NC, miR-26a mimic or anti-miR-26a inhibitor for 48 h, then subjected to TMZ treatments (250 µM, 72 h). Cell death ratio was detected by ANXA5 and PI staining. The apoptotic and secondary necrotic cells was shown as the sum of ANXA5-positive and ANXA5 and PI double-positive cells. Results were quantified as the percentage of apoptosis ANXA5-positive and ANXA5 and PI double-positive cells in total cells and presented in the right panel. **d** Caspase-3, cleaved caspase-3, PARP, cleaved PARP protein levels were detected in the stably expressing miR-NC, miR-26a, or anti-miR-26a cell lines. **e** Western blot analysis showed cytochrome *C* levels of cytoplasm and mitochondria in the stably expressed miR-NC, miR-26a, or miR-26a-sponge cell lines after TMZ treatments (250 µM, 72 h). β-actin and VDAC levels were served as the internal control of cytoplasmic and mitochondrial proteins, respectively. **f** U87MG cells transduced with miR-NC, miR-26a mimics or anti-miR-26a inhibitor after treatment with TMZ (250 µM) for 12 h was stained with JC-1 probe and imaged by fluorescent microscope. Scale bar = 100 μm. **g** U87MG/miR-NC, U87MG/miR-26a, and U87MG/ anti-miR-26a cells (1 × 10^5^ cells) were dispersed in 5 μL of Matrigel and were implanted intracranially in each mouse. Representative pseudocolor bioluminescence images of intracranial mice bearing miR-NC, miR-26a, or anti-miR-26a transduced U87MG cells after treatment with TMZ at indicated time points. TMZ treatment (20 mg/kg) group: *n* = 12 for miR-NC, *n* = 10 for miR-26a, *n* = 10 for miR-26a-sponge. **h** Intracranial tumors in brain were shown by T2-weighted MRI and H&E staining. Scale bar = 1 mm. **i** Immunohistochemical (IHC) staining of tumor sections for cleaved caspase-3 signals. Scale bar: 100 μm. **j** Kaplan–Meier curves were drawn to measure the survival abilities of U87MG stable cell lines. Data were presented by means ± SEM in triple experiments. Asterisk or hash indicated significant difference at *p* < 0.05 compared with miR-NC or anti-miR-NC

To analyze the role of miR-26a in glioma chemo-resistance to TMZ treatment, U87MG cells were treated at various TMZ concentrations. As Fig. 3a, compared with control group, miR-26a overexpression significantly suppressed the chemo-sensitivity to TMZ. However, anti-miR-26a inhibitor increased the chemo-sensitivity of the cells to TMZ treatment. Similarly, inhibition of miR-26a using anti-miR-26a inhibitor in U87MG cells significantly reduced growth rate and the ability to form a colony. Overexpression of miR-26a promoted cell growth and colony formation (Fig. 3b and S3a). In order to determine whether miR-26a plays a key role in cell apoptosis in the presence of TMZ treatment, overexpression of miR-26a decreased the percentage of annexin V-positive cells, whereas anti-miR-26a inhibitior treatment increased the cell apoptosis rate with a concomitant TMZ treatment (Fig. 3c). Furthermore, we examined the expression levels of various apoptotic components and found that miR-26a overexpression led to the inhibition of cleaved caspase-3 and cleaved PARP, and anti-miR-26a inhibitor increased expression levels of those apoptotic associating proteins (Fig. 3d). Meanwhile, to evaluate whether miR-26a has an effect on the DNA damage and cell death after TMZ treatment, our results showed that miR-26a may not affect the expression of γH2AX in response to TMZ treatment (Fig. S3b, c). Intriguingly, we found that miR-26a inhibition induced transposition of cytochrome *C* in mitochondria to cytoplasm for inducing cell apoptosis (Fig. 3e). Then, the image using JC-1 probe by fluorescent microscope showed that TMZ induced mitochondrial depolarization and cells changed from red to green in both control groups as we showed in Fig. 1e before. MiR-26a mimics increased the red/green fluorescence intensity ratio and anti-miR-26a inhibitor decreased that ratio, suggesting that miR-26a protects mitochondrial function (Fig. 3f). To further explore the effect of miR-26a on TMZ resistance in vivo, tumor growth assay was performed and monitored using pseudocolor bioluminescence images of intracranial mice after TMZ treatment for 14 days. The results showed that miR-26a overexpression made U87MG cells to increase chemo-resistance and activity of bioluminescence, while anti-miR-26a inhibitor enhanced chemo-sensitivity and decreased activity of bioluminescence (Fig. 3g). T2-weighted MRI and hematoxylin and eosin staining confirmed this finding that tumors with miR-26a-overexpression were bigger than those with miR-NC, while anti-miR-26a inhibitor drastic attenuated tumor growth (Fig. 3h). We found that miR-26a-overexpressing tumors showed a lower apoptotic index as demonstrated by active caspase-3 staining compared with control tumors (Fig. 3i). Kaplan–Meier curves analysis showed that mice bearing miR-26a overexpression tumors had a shorter survival than those bearing anti-miR-26a inhibitor group (Fig. 3j). Thus, our results demonstrate that miR-26a promotes TMZ resistance of glioma cells by inhibiting apoptosis and protecting mitochondrial membrane integrity.

### Bax and Bad are direct targets of miR-26a

**Fig. 4:**
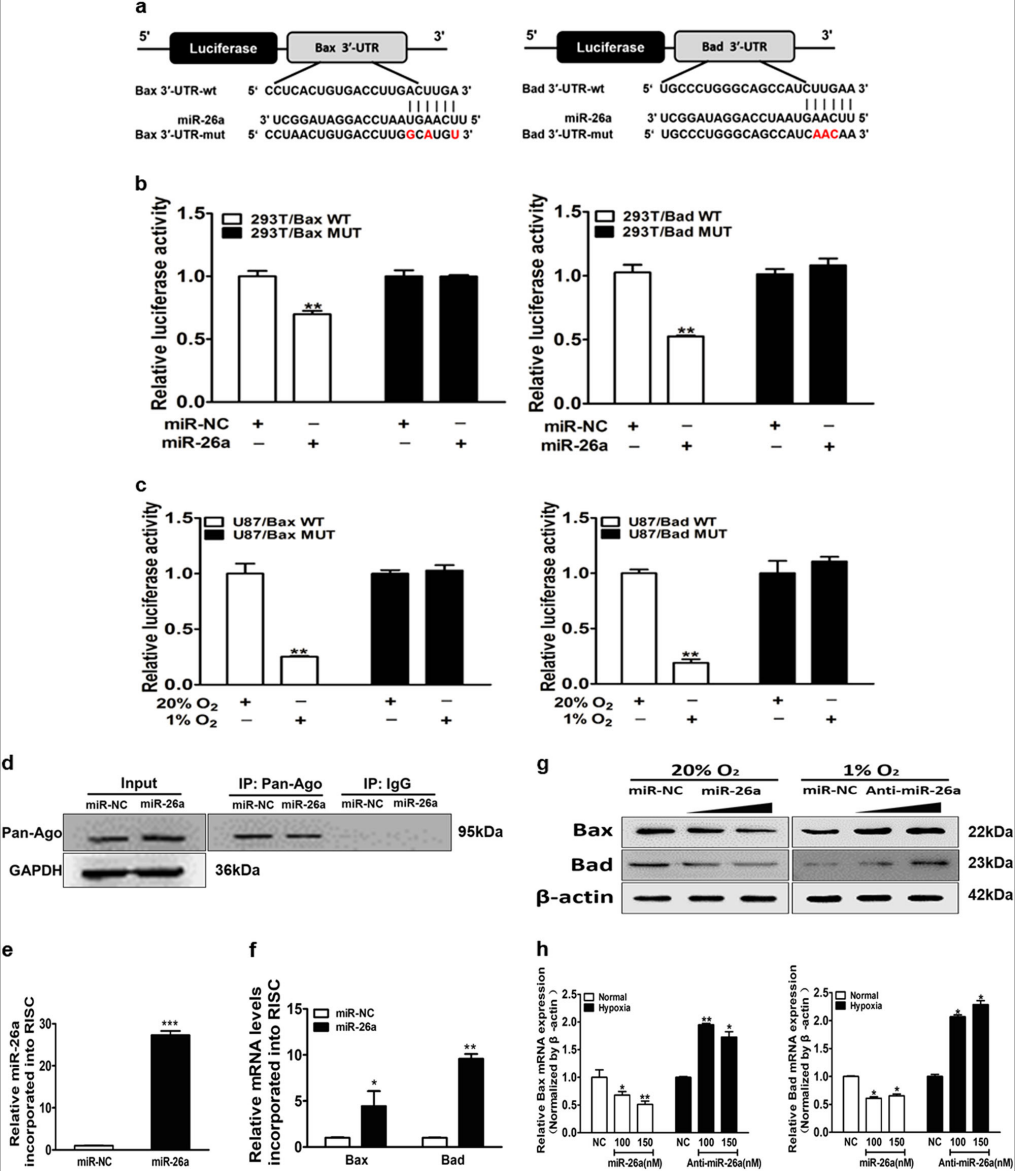
Bax and Bad are direct targets of miR-26a. **a** The complementary pairing of miR-26a with Bax and Bad wild-type (WT) and mutant (Mut) 3′-UTR reporter constructs were shown. **b** The reporter plasmids carrying the WT or Mut Bax and Bad 3′-UTR regions were co-transfected with miR-NC, miR-26a mimics and pRL-TK into 293T cells. After 24 h of the transfection, the relative luciferase/pRL-TK activities were analyzed. **c** WT or Mut 3′-UTR constructs of Bax and Bad were transfected into U87MG cells, then exposed to normoxia or hypoxia for 24 h. The relative luciferase/pRL-TK activities were determined. **d** Immunoblotting was used to detect the Ago2-RISC complex by the Ago2 antibody from U87MG cells with miR-NC or miR-26a mimics. Negative control was IgG. GAPDH was used as an internal control. **e** qRT-PCR was conducted to measure miR-26a levels incorporated into RISC in cells overexpressing miR-26a and miR-NC. U6 RNA levels were used as an internal control. **f** qRT-PCR analysis was performed to measure levels of indicated Bax and Bad mRNA levels incorporated into RISC derived from miR-26a or miR-NC cells. GAPDH levels were used as an internal control. **g**, **h** U87MG cells exposed to normoxia or hypoxia were transfected with miR-26a mimics (100 and 150 nM) or anti-miR-26a inhibitor (100 and 150 nM). Bax and Bad expression levels were determined by immunoblotting and qRT-PCR using β-actin as an internal control. Data are presented as mean ± SEM from three independent experiments. Asterisk indicates significant difference at *p* < 0.05 compared with control group and double asterisk indicates significant difference at *p* < 0.01 compared with control group

To identify the mechanism of miR-26a in conferring TMZ resistance, we predicted its potential downstream targets using algorithmic programs (TargetScan and MiRanda). Bcl-2 family genes, Bax and Bad are two candidate targets of miR-26a based on the pairing of the seed sequence of miR-26a (Fig. 4a). To determine whether miR-26a regulates Bax and Bad expression through binding to their 3′-UTR, we made the wild (WT) or mutant (Mut) luciferase reporter plasmids containing the potential binding site of Bax and Bad 3’-UTR and performed luciferase assay. As shown in Fig. 4b, miR-26a decreased the luciferase activities of WT plasmids, but not the Mut plasmids. Further study showed that the luciferase activities of WT plasmids were reduced under hypoxic condition compared with normoxic condition in U87MG (Fig. 4c). To confirm Bax and Bad are direct targets of miR-26a, we identified if mRNAs of Bax and Bad selectively enriched in the Ago2/RISC complex after miR-26a overexpression using RNA-ChIP analysis (Fig. 4d). As an internal positive control, miR-26a incorporation into RISC was significantly increased after transfection of miR-26a (Fig. 4e). As expected, Bax and Bad significantly elevated enrichment in miR-26a-overexpressing cells compared with the miR-NC group (Fig. 4f). The mRNA and protein levels of Bax and Bad were inhibited by miR-26a mimics under the normoxia condition, and increased by anti-miR-26a inhibitor under the hypoxia condition (Fig. 4g, h). Furthermore, consistent with the time-dependent increase of miR-26a upon hypoxia, hypoxia also decreased the expression levels of Bax and Bad in a time-dependent manner (Fig. S4a-b). The results demonstrate that Bax and Bad are direct targets of miR-26a.

### Bax and Bad are key targets of miR-26a in TMZ resistance

**Fig. 5:**
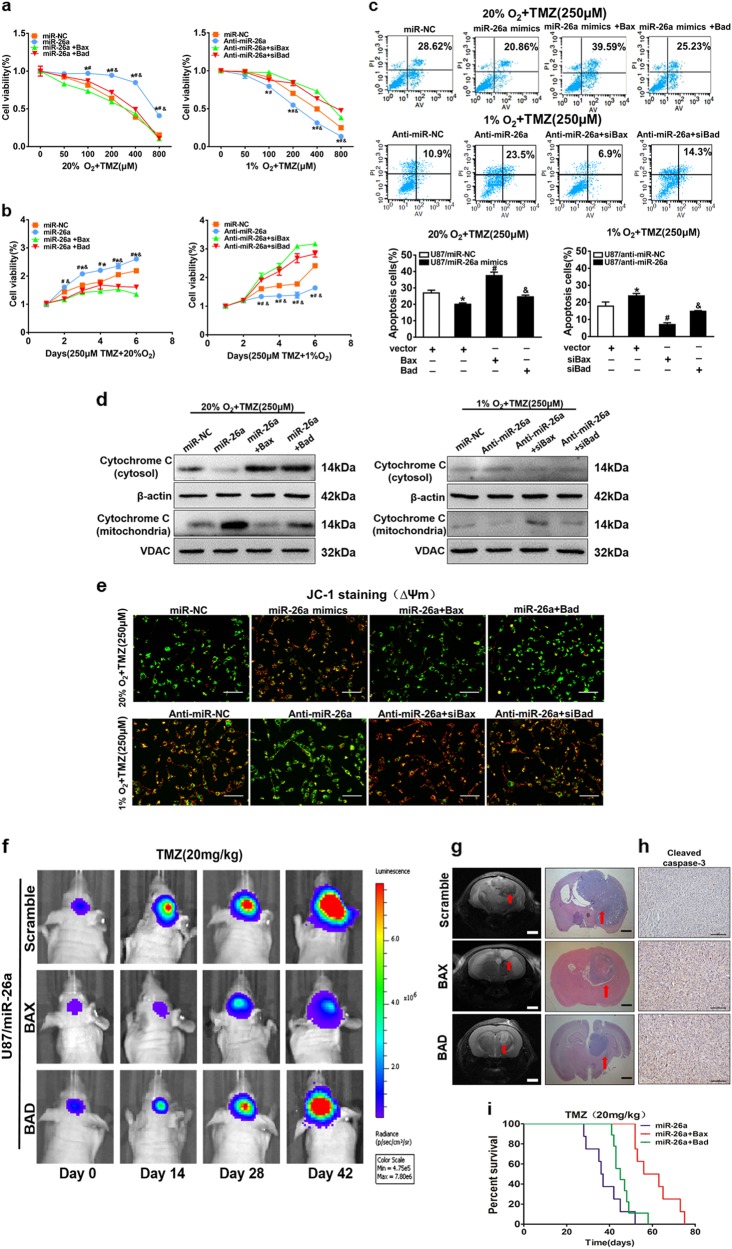
MiR-26a/Bax/Bad axis contributes to hypoxia-mediated TMZ resistance in vitro and in vivo. **a** Cell viability was analyzed by CCK8 assay 48 h after TMZ treatments as indicated. **b** Cell viability was tested every 24 h for different times with treatment of 250 µM TMZ. **c**, **e** Cell apoptosis and mitochondrial membrane potential function were evaluated by flow cytometry and JC-1 probe kit in U87MG cells with co-transfection miR-NC or miR-26a mimics, with or without overexpression of Bax and Bad under normal condition. Meanwhile, U87MG cells with co-transfection anti-miR-NC or anti-miR-26a mimics with or without siBax and siBad under hypoxic condition. U87MG cells exposed to normoxic or hypoxic condition were pretreated with indicated TMZ treatments (250 μM), then subjected to apoptosis assay. Scale bar = 100 μm. **d** Western blot analysis showed expression levels of cytochrome *C* in mitochondria and cytoplasm using stably expressing miR-NC, miR-26a, or anti-miR-26ainhibitor with indicated treatment. **f** Representative pseudocolor bioluminescence images of intracranial mice bearing miR-26a with or without the overexpression of Bax and Bad upon TMZ treatments. Cells were implanted intracranially in each mouse. 10 mouse each group. **g** T2-weighted MRI and H&E staining of brain sections showed intracranial tumors. Scale bar = 1 mm. **h** IHC staining of cleaved caspase-3. Scale bar = 100 mm. **i** Kaplan–Meier curves were drawn to measure mouse survival with the tumor burden induced by U87MG cells stably expressed miR-26a, with or without the overexpression of Bax and Bad. Data are presented as mean ± SEM from three independent experiments. Asterisk indicates significant difference at *p* < 0.05 compared with control group, hash indicates significant difference at *p* < 0.05 compared with control group and ampersand indicates significant difference at *p* < 0.05 compared with control group

To determine the role of Bax and Bad in hypoxia-induced TMZ resistance, Bax and Bad expression vector and their small interference RNAs were firstly transfected into U87MG cells, and western blotting results confirmed high transfection efficiency (Fig. S5a). Then CCK8 proliferation assay showed that overexpression of Bax and Bad in U87MG cells decreased miR-26a-induced resistance to TMZ under normoxia, whereas knockdown of Bax and Bad in U87MG cells increased anti-miR-26a-suppressed sensitivity to TMZ under hypoxia (Fig. 5a). Furthermore, cell growth rate, DNA synthesis rate (EdU assay) and colony formation ability in the presence of TMZ (250 μM) under normoxic or hypoxic conditions were assayed. We found that forced expression of Bax or Bad partially abolished miR-26a-induced cell resistance to TMZ treatment, the higher ability of colony formation and DNA synthesis rate under normal condition. Under hypoxia, siBax or siBad restored anti-miR-26a-suppressed chemo-resistance to TMZ, colony formation ability and DNA synthesis rate in U87MG cells (Fig. 5b and S5b, d). To further assess whether miR-26a, Bax and Bad involved in cell apoptosis in the presence of TMZ under normoxic or hypoxic conditions, FACS analysis revealed that overexpression of miR-26a inhibited TMZ-promoted apoptosis activity under normoxia condition, which was partially reversed by Bax or Bad overexpression. Also, anti-miR-26a inhibitor promoted TMZ-induced apoptosis under hypoxia condition, which was suppressed by siBax or siBad (Fig. 5c). Next, after TMZ treatment, Bax or Bad overexpression in U87MG/miR-26a increased cytochrome *C* levels in cytoplasm and reduced that in mitochondria under normoxia. In U87MG/anti-miR-26a cell line, siBax or siBad partly reversed the effect induced by miR-26a inhibition (Fig. 5d). Furthermore, mitochondrial functions were detected by JC-1 assay and immunocytochemistry. Forced expression of Bax and Bad increased miR-26a overexpression-decreased mitochondrial membrane potential disruption, whereas knockdown of Bax and Bad restored anit-miR-26a inhibitor-induced mitochondrial membrane potential disruption upon TMZ treatment under the normoxia or hypoxia condition, respectively (Fig. 5e). Mitotracker was used for mitochondria tracking and cytochrome *C* was the target protein (Fig. S5c). Thus, we discovered that miR-26a/Bax/Bad axis contributes to hypoxic-mediated TMZ resistance by influencing cytochrome *C* location, mitochondria function, and apoptosis.

To further evaluate the role of miR-26a/Bax/Bad axis on TMZ resistance in vivo, the cells combination of miR-26a or Bax /Bad were implanted intracranially in each mouse and treated by TMZ at day 14 after implantation and representative pseudocolor bioluminescence images of intracranial mice were conducted. The results showed that Bax/Bad overexpressiondecreased activities of bioluminescence and inhibited tumor growth, further confirmed that Bax/Bad overexpression promotes TMZ sensitivity of glioma cells in vivo (Fig. 5f). T2-weighted MRI and hematoxylin and eosin staining confirmed that mice bearing Bax/Bad overexpression produced smaller tumor than miR-26a (Fig. 5g). We also found that Bax/Bad overexpression tumors showed a higher apoptotic index as demonstrated by active caspase-3 staining compared with miR-26a group (Fig. 5h). Kaplan–Meier curves were drawn to measure mouse survival with tumor burden induced by U87/miR-26a with or without Bax/Bad overexpression, and U87MG/miR-26a with Bax/Bad overexpression showed a higher survival rate (Fig. 5i). Taken together, our study first demonstrates that miR-26a/Bax/Bad axis contributes to hypoxic-mediated TMZ resistance in vitro and in vivo.

### MiR-26a overexpression strongly correlates with HIF-1α, Bax, Bad levels, and poor prognosis in GBM receiving temozolomide therapy

**Fig. 6:**
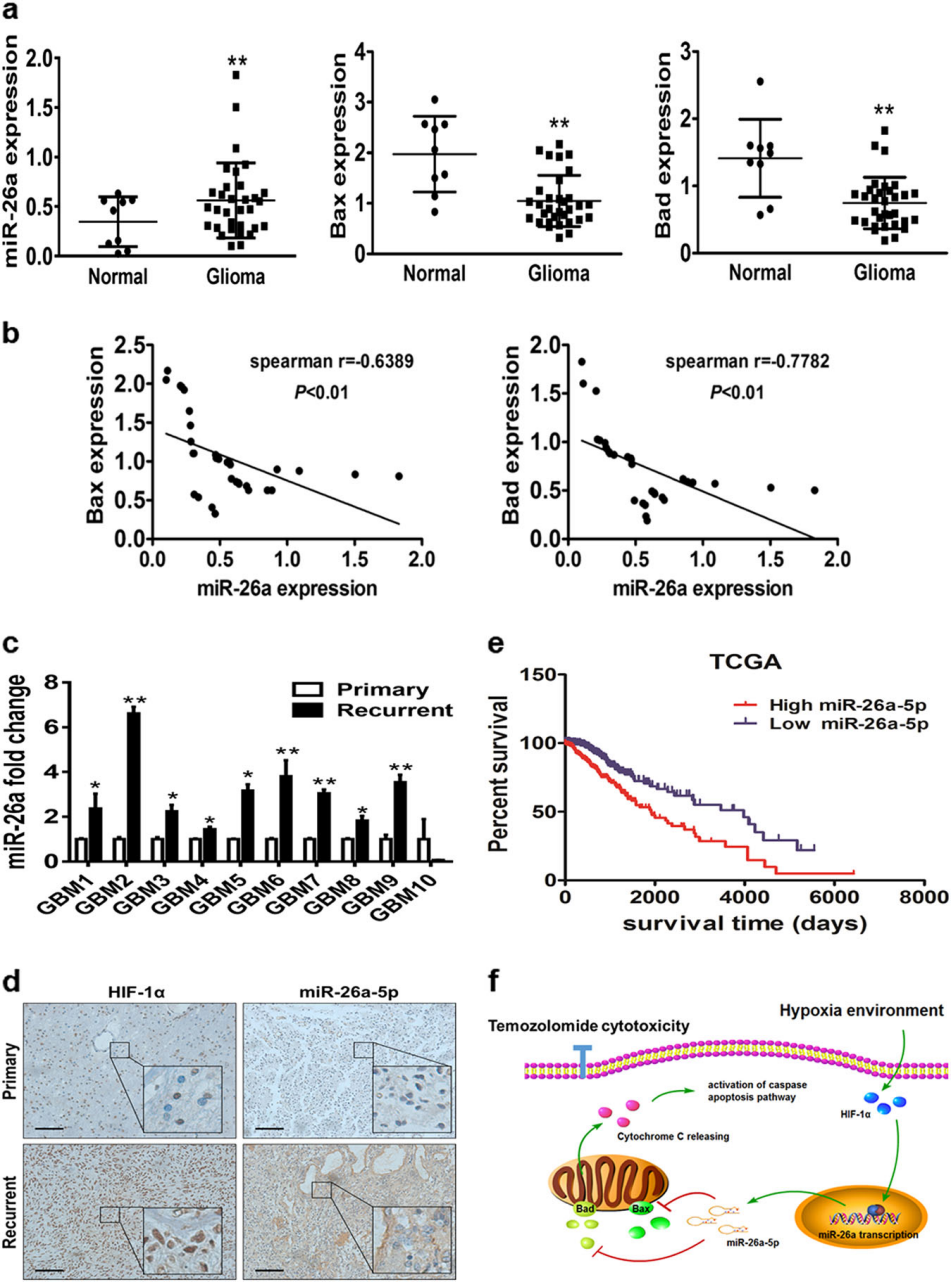
MiR-26a overexpression positively correlates with HIF-1α, Bax, and Bad levels, and poor prognosis in GBM receiving temozolomide therapy. **a** The expression levels of miR-26a, Bax and Bad in normal brain and glioma tissues. **b** Spearman′s correlation analysis determined the correlation between expression levels of Bax, Bad, and miR-26a in human GBM specimens. **c** qRT-PCR analysis of miR-26a expression levels in primary and recurrent glioma tissues from 10 patients who are treated with TMZ therapy regularly. **d** Representative images of IHC staining of HIF-1α and ISH staining of miR-26a expression in a pair of primary and recurrent GBM tumors (×200 and ×400 magnification). Scale bar: 100 μm. **e** Based on TCGA public datasets, Kaplan–Meier curves showed the negative correlation between miR-26a and clinical outcome. **f** The diagram summarizes our findings: hypoxia induces activation of miR-26a by HIF-1α. HIF-1α/ miR-26a axis rescues Bax/Bad-driven mitochondrial membrane dysfunction and subsequently, suppresses cytochrome *c* release and activation of caspase-3, which helps the GBM cell survive under TMZ treatment. Asterisk indicates significant difference at *p* < 0.05 compared with primary glioma tissue or normal tissue and double asterisk indicates significant difference at *p* < 0.01 compared with primary glioma tissue or normal tissue

To explore functional relevance of miR-26a and its targets in clinic samples, we examined the expression of miR-26a, Bax, and Bad mRNA in 9 normal brain tissues and 32 human glioma specimens. Our results demonstrated that the mRNA expression levels of Bax and Bad were downregulated in glioma specimens while miR-26a was upregulated (Fig. 6a). Spearman’s correlation analysis determined that Bax, Bad levels were inversely correlated with miR-26a levels in human glioma specimens (Fig. 6b). To evaluate the clinical relevance of miR-26a and prognosis of GBM patients, 10 matched pairs of primary GBM and recurrent TMZ-refractory GBM were analyzed by qPCR. The results confirmed that miR-26a levels were significantly upregulated in the recurrent tumor samples by contrast to their respective primary tumor tissues (Fig. 6c). Moreover, the immunohistochemistry (IHC) and in situ hybridization (ISH) demonstrated that the recurrent tumors exhibited significantly higher HIF-1α and miR-26a staining intensity compared with primary tumors on the same set of GBM tissue (Fig. 6d). TCGA database analysis showed that high expression levels of miR-26a were consistently correlated with the poor clinical outcome of GBM patients (Fig. 6e). Finally, we summarized an ideograph to illustrate the crucial role of the HIF-1α/miR-26a/Bax/Bad pathway on TMZ resistance process (Fig. 6f). These results support the notion that a hypoxia-miR-26a signaling pathway has an important role under hypoxia-induced GBM chemo-resistance.

## DISCUSSION

Hypoxia is a common microenvironment among different kinds of cancer tissues including GBM. Hypoxia exerted crucial effects on different cellular and physiologic processes including cell proliferation, apoptosis, metabolism, and angiogenesis^27–29^. Moreover, tumor hypoxia is associated with resistance to chemotherapeutic agents as well as radiotherapy, ultimately limiting patient prognosis^30^. In this study, we firstly demonstrated that hypoxia improved tolerance of GBM cells to TMZ and promoted tumor cells survival though decreasing DNA damage and protecting mitochondria function. Thus, it is becoming more important to elucidate the mechanism of TMZ resistance in hypoxic conditions for GBM. Emerging studies have shown that several miRNAs were dysregulation during hypoxic conditions in cancer cells, such as miR-210, miR-630, and miR-421^31–33^. Previously, we showed that miR-26a expression levels were higher in glioma species and promoted tumor growth and angiogenesis in glioma. Here, we found a novel role of miR-26a to enhance TMZ resistance of glioma cells in vitro and in vivo models. Intriguingly, we also found that miR-26a was upregulated by HIF-1α as the pivotal molecular regulator of oxygen homeostasis under hypoxic conditions.

TMZ is the GBM first-line therapy in clinic; however, patients who initially respond to therapy often develop chemo-resistance to TMZ rapidly. The hypoxic microenvironment as one of the main factors induced resistance to TMZ by activating targets in the mammalian target of rapamycin (mTOR) pathway and BIRC3 expression, but specific mechanism is still not clear^34^. Generally, TMZ fights against cancer cells by DNA damage and follow-up apoptosis through the intrinsic pathway, which is also known as mitochondria-dependent apoptosis^35,36^. A series of studies illustrates the mechanism of apoptosis event by several protein families; specifically the upstream Bcl-2 family (e.g., Bcl-2, Bax, and Bad) and the downstream caspase family (e.g., caspase-3)^37^. According to this study, Bcl-2 prevents cytochrome *c* release and caspase activation, which in turn promotes cytochrome *c* release into the cytosol from mitochondria and activated cascade activation of caspase activities^38^. The studies have shown that treatment with TMZ changes pro-apoptotic Bax and anti-apoptotic Bcl-2 expression levels involved in the mitochondrial pathway of apoptosis^39^. However, the mechanism how Bcl-2 family expression is driven by in hypoxia-induced TMZ resistance in glioma still remains unclear. Our study identified that Bax and Bad were direct and functional targets of miR-26a and emerged the clinical relevance, evidenced by the inverse correlations between miR-26a and Bax/Bad in clinical tissues. Our further studies using animal and human tumor tissues suggests that miR-26a/Bax/Bad axis is important in acquired TMZ resistance in glioma hypoxia microenvironment.

Numerous studies showed that miR-26a had different roles depending on tumor and tissue types, for example, suppressor gene in lung cancer, melanoma, and breast cancer, while oncogene in glioma^40^. In this study, the TCGA database also confirmed that high expression of miR-26a correlated with poor survival in patients with GBM, consistent with other studies. In recent years, the contribution of HIF-1 to drug resistance has been observed in diver tumor^40^. There new findings predict that miR-26a expression levels could be used to indicate therapeutic response to TMZ in GBM. And one of interesting studies found that miR-26a levels were up-regulated in lymph node metastasis tumor tissues in contrast to primary tumor tissues, enhanced metastasis potential of lung cancer cells^41^. It is well known that lung cancer has a higher incidence of metastasis to the brain, and TMZ combined with other therapies may have a better effect on patients with brain metastasis from lung cancer. Although the mechanism for the difference between the primary tumor and metastases of lung cancer is not clear, miR-26a should be further investigated as a potential prognostic biomarker and therapeutic target for TMZ chemotherapy resistance.

In summary, our results demonstrate that miR-26a is an important regulator of TMZ resistance induced by hypoxia, which can effectively protect mitochondria function and reduce apoptosis by targeting bax and bad. Our study significantly broadens the understanding of miR-26a in chemotherapy resistance. It is interesting to evaluate whether targeting miR-26a may have potential value as an adjuvant therapy in the future.

## MATERIALS AND METHODS

### Cell lines and reagents

U87MG and HEK293T cells were obtained from American Type Culture Collection and were cultured in Dulbecco’s Modified Eagle Medium (Gibco, Grand Island, NY, USA) with 10% FBS. Temozolomide (TMZ) was purchased from Selleck Chemicals (Shanghai, China). Echinomycin was purchased from Sigma (St. Louis, MO, USA). Bevacizumab was purchased from Roche (Welwyn Garden City, UK). See Supplementary Table 1 for detail information of antibodies.

### Lentiviral packaging and stable cell line establishment

Lentivirus expressing miR-26a, miR-NC (negative control) and anti-miR-26a inhibitor were purchased from Genepharma (Shanghai, China). Stable cell lines U87/miR-NC, U87/miR-26a, and U87/anti-miR-26a were established by infecting U87 cells with lentivirus, then selected using puromycin (Sigma, MI, USA).

### Cell proliferation and colony formation assay

To confirm miR-26a effect on TMZ treatment, we planted 3000 cells per well in 96-well plates. The absorption value was measured by CCK8 kit (Dojindo Laboratories, Kumamoto, Japan) at different time points indicated. Data were obtained from three separate experiments with six replications each time.

For colony formation assays, cells were placed in 24-well plates with 200 cells per well. The medium was changed every 4 days. Cells were fixed with methanol 14 or 28 days later, stained with 0.05% crystal violet, photographed, and counted. We did triplicate for each group. And experiments were independently repeated three times.

### EdU proliferation assay

Cell-Light™ EdU Apollo®488 In Vitro Imaging Kit (Ribobio, Guangzhou, China) was performed as manufracture instruction to determine cells proliferation. Cell nuclei were stained for 30 min by Hoechst 33342 at 5 µg/mL. After incorporation, a fluorescent dye making possible fluorescent visualization of proliferating cells was used that reacted specifically with EdU. Microscopic observation was performed under a fluorescence microscope (ZEISS, Germany).

### Dual-luciferase reporter assay

For dual-luciferase assay, Bad or Bax 3′-UTR region containing putative miR-26a matching sites (wide type, WT) and corresponding mutant sites (Mut) were cloned into the *Sac*I and *Hin*dIII restriction enzyme sites of pMIR-REPORTER vector (Ambion, CA, USA), immediately downstream of luciferase stop codon. Then we transfected the Wild-type constructs and mutant ones into the cells and co-transfected luciferase reporter plasmids containing either wild-type (WT) or mutant-type (Mut) of Bad or Bax -3′UTR regions, pGL4.74 luciferase reporter vector (Ambion) and miR-26a mimics or miR-NC mimics, anti-miR-26a inhibitor or anti-miR-NC inhibitor (Thermofisher, US) using jetPRIME into U87 cells. Firefly and Renilla luciferase activities were confirmed using a dual-luciferase assay kit (Promega, WI, USA) at 24 h after transfection. Primers used for reporter constructions were shown in Supplemental Table S2.

### RNA isolation and quantitative real-time PCR (qPCR)

Total RNAs of cells were extracted using TRIzol reagent (Takara, Dalian, China) from cultured cell or fresh tissues following the manufacturer’s instructions. qPCR was conducted to test expression of miR-26a and mRNA. U6 and GAPDH was used as an endogenous control. qPCR using SYBR Premix DimerEraser (Takara, Dalian, China) was performed on the 7900HT system. Relative expression levels of the target gene were determined by the comparative cycle threshold method (2^−ΔΔCt^). Primers used for qPCR were shown in Supplemental Table S1.

### Protein extraction and western blotting

RIPA buffer (150 mM NaCl, 50 mM Tris, pH 7.4, 1% Triton X-100, 1 mM EDTA) was used to lyse cells on ice for 30 min. The lysates were centrifugated at 13,000 rpm 4 °C for 15 min, and the supernatants were collected and protein concentrations were determined using BCA assay (Beyotime Institute of Biotechnology, China). Samples were separated by SDS-polyacrylamide gel electrophoresis (SDS-PAGE) and transferred to PVDF membranes in transfer buffer. Membranes were blocked with 5% nonfat milk in 1 × PBS containing 0.05% Tween-20 and incubated overnight with primary antibodies at 4 °C. The protein bands were detected with appropriate horseradish peroxidase conjugated secondary antibodies and reacted with the SuperSignal West Pico Chemiluminescent Substrate Kits (Thermo Scientific, MA, USA). Antibodies used for western blotting were shown in Supplemental Table S2

### Flow cytometry analysis

Cells were treated with TMZ as indicated. ANXA5 and PI was stained by the Alexa Fluor 488Annexin V Dead Cell Apoptosis Kit (BD Pharmingen) as protocol. The cells were analyzed by flow cytometry (BD Bioscience, FACSCalibur, Singapore), using 488 nm excitation and measuring the fluorescence emission at 530 and 585 nm. FlowJo software were used to analyze data. Three experiments were performed in triplicate.

### Chromatin immunoprecipitation (ChIP) assay

ChIP assays were performed using the Simple ChIP PlusEnzymatic Chromatin IP Kit (Agarose Beads) (Cell Signaling Technology, USA). Cells were cross-linked by addition of 37% paraformaldehyde at 37 ℃ for 20 min and quenched by glycine. Then cells were rinsed with PBS buffer. The extracted chromatin was digested and fragmented into 150 to 900 bp, then DNA was enriched from the cell lysates by specific antibodies immunoprecipitating HIF-1α or IgG overnight at 4 °C, and collected by incubation with Protein A/G Agarose/Salmon Sperm DNA (50% Slurry) for 2 h. The purified DNA was used as a template for qPCR. Fold enrichment was analyzed according to Ct as 2-Δ(ΔCt), where ΔCt = CtIP-CtInput and Δ(ΔCt) = ΔCtantibody-ΔCtIgG. Sequences of Primers used in this study are listed in Supplementary Table S2.

### Isolation of RISC-associated RNA

U87MG cells transfected with miR-NC or miR-26a were fixed with 1% formaldehyde, followed by chromatin fragmentation. Cells were lysed in NETN buffer and incubated with Dyna beads Protein A (Invitrogen) together with anti-pan Ago antibody (Millipore) or IgG control for immunoprecipitation. After proteinase K digestion and extraction, the immunoprecipitated RNA was acquired. Ethanol with glycogen was used to precipitate RNA.

### Measurement of mitochondrial membrane potential (ΔΨm) by fluorescence microscopy

The mitochondrial membrane potential was analyzed by fluorescence microscopy using the 5,50,6,60-tetrachloro-1,10,3,30-tetraethylbenzimidazol carbocyanineiodide probe. After treatment as indicated, we mixed JC-1 staining solution (Beyotime, China) and culture medium at the ratio of 5 μL per millilite and incubated the samples in cell culturing incubator for 20 min avoided from light. After twice washing, cells were checked using a fluorescence microscope (Zeiss, Germany).

### Immunofluorescence assay

Cells were treated using the procedure as indicated. Then cells were incubated with 50 nM MitoTracker Green to visualize mitochondria. Cells were fixed 30 min later with 37% paraformaldehyde and permeabilized with PBS containing 0.5% Triton X-100 at room temperature. And 1% bovine serum albumin (BSA) in PBST was used to block the samples. Bax and Bad antibodies was used for immunostain overnight at 4 °C. The fluorescence isothiocyanate (FITC)-labeled goat anti-rabbit secondary antibody (Santa Cruz) and tetramethylrhodamine isothiocyanate (TRITC)-labeled goat antimouse secondary antibody (Santa Cruz) were incubated with the samples. Cells were incubated with 10 μM Hoechst 33342 for 10 min to visualize nuclei. Microscopic observation was performed under a fluorescence microscope (ZEISS, Germany).

### In situ hybridization

Paraffin-embedded tissues were sectioned at 5 μm and then deparaffinized in xylenes. After above processes, tissue slides were rehydrated through an graded ethanol dilution series (from 100 to 70%). Slides were submerged in diethylpyrocarbonate-treated water and subjected to Hydrogen Peroxide treatment and Pepsin digestion, refixed in 4% paraformaldehyde, then slides were rinsed with PBS between treatments. Slides were prehybridized in hybridization solution at 38–42 °C for 60 min. Then, 10 pmol probes (Boster, Wuhan, China) complementary to miR-26a was added and hybridized for 20 h at a temperature of 38–42 °C following washes in SSC at the same temperature. After incubating with anti-DIG-HRP Fab fragments conjugated to horseradish peroxidase, the hybridized probes were measured by 3′-3-diaminobenzidine solution, and nuclei were counterstained with Haematoxylin.

### Immunohistochemistry assay

Fresh tumor tissues were fixed with Bouin solution for 24–48 h and processed by the paraffin-embedded method. Deparaffinage and dehydration were as same as the processes of in situ hybridization. Following, tissue slides were heat immobilized or pepsin immobilized and incubated with antibody against HIF-1α or Caspase-3 overnight at 4 ℃. After incubated with secondary antibody, the slides were reacted with DAB Histochemistry Kit (Invitrogen, USA).

### In vivo study

Female BALB/c nude mice, 4 to 5 weeks old, were purchased from Vital River Animal Center (Beijing, China) and maintained in special pathogen-free (SPF) condition for one week. Animal experimental procedures were in consistent with the Care and Use of Laboratory Animals Guide and approved by the Animal Experimental Ethics Committee of Nanjing Medical University. Cells (4 × 10^6^) mixing with 150 μL of FBS-free DMEM medium were subcutaneously injected into both sides of the posterior flanks of 5 nude mice per group. After implantation for 24 days, mice were killed to dissect the tumor tissues. Total proteins and RNAs were extracted for further detection. Tumors were formalin-fixed, paraffin-embedded, and tumor sections at 5 μm were analyzed by immunohistochemical staining for HIF-1α levels and by hybridization in situ for miR-26a levels.

U87-Luc cells (1 × 10^5^) with indicated virus infection or treatment were resuspended in Matrigel in a total volume of 5 μL and injected intracranially into the striatum of adult nude mice (6-week-old) by a stereotactic device (coordinates: 2 mm anterior, 2 mm lateral, 3 mm depth from the dura). At one week after injection, the tumor-bearing mice were intraperitoneally injected with 20 mg/kg TMZ in saline (final DMSO concentration 25%). To visualize the injected cells, mice were performed intraperitoneal injection with D-luciferin (150 mg/kg) and formated of image under anesthesia with the IVIS Illumina System (Caliper Life Sciences). Picture acquisition was conducted once a week to supervise tumor growth. After 6 or 8 weeks, brains were perfused with 4% paraformaldehyde by cardiac perfusion and further fixed with Bouin solution at 4 °C overnight. Then brains were resected and performed H&E stainings.

### Statistical analysis

Data shown in our study were represented as means ± SD from at least three times independent experiments. Student’s unpaired *t*-test was conducted for comparison between two groups. Significantly difference were considered when *p* < 0.05.

## Electronic supplementary material

Supplement Figure legend

supplemental-table1

supplemental-table2

Supplement_Fig1

Supplement_Fig2

Supplement_Fig3

Supplement_Fig4

Supplement_Fig5-1

Supplement_Fig5-2

Supplement_Fig5-3

Supplement_Fig5-4
