# Supplementary material for: Hypoxia-mediated mitochondria apoptosis inhibition induces temozolomide treatment resistance through miR-26a/Bad/Bax axis
Source: Cell Death Dis. 2018 Nov 13;9(11):1128. doi: 10.1038/s41419-018-1176-7 (PMC6233226; doi:10.1038/s41419-018-1176-7)
Supplement: Supplementary file 1 — Supplement Figure legend [file 41419_2018_1176_MOESM1_ESM.docx]

**Figure legends of supplementary**

**Supplementary Figure 1 Hypoxic microenvironment improves tolerance of glioma cells to temozolomide though decreased DNA damage and protection mitochondria function. (a)** U87MG cells were exposed to hypoxia (1% O_2_) for different time points as indicated. The protein levels of HIF-1α were analyzed by immunoblotting. β-actin levels served as the internal control. **(b)** Colony formation ability of U87MG cells cultured under normoxic or hypoxic condition in the absence or presence of TMZ. **(c)** EdU assay of U87MG cells exposed to normoxic or hypoxic condition plus treatment with DMSO or TMZ (250μM) for 72h were conducted. Scale bar= 100 μm. Data were presented by means ± SEM. in triple experiments. * indicated significant difference at *P*<0.05 compared with normoxia. ** indicated significant difference at *P*<0.01 compared with normoxia.

**Supplementary Figure 2 MiR-26a expression is up-regulated under hypoxia through HIF-1α in glioma cells. (a)** Quantitative real-time PCR was used to detect the differential expression miRNAs in glioma under hypoxic conditions. **(b)** Quantitative real-time PCR was performed to measure time-dependent expression levels of miR-26a after short exposure to hypoxia for 0, 2, 4 or 6 h. **(c)** Expression levels of CTDSPL and CTDSPL2 were examined in U87MG cells treated as indicated. **(d, e).** CTDSPL and CTDSPL2 levels were measured in cells overexpression or interference of HIF-1α**.** Data were presented by means ± SEM. in triple experiments. * indicated significant difference at *P*<0.05 compared with control group. ** indicated significant difference at *P*<0.01 compared with control group.

**Supplementary Figure 3 MiR-26a conferred glioma cells resistance to TMZ. (a)** Colony formation ability of the U87MG stably expressing miR-NC, miR-26a or miR-26a-sponge cells in the presence of TMZ. **(b)** Western blot analysis showed γ-H2AX levels in the stably expressed miR-NC, miR-26a or miR-26a-sponge cell lines after TMZ treatments (250 µM, 72 h). **(c)** Cyto-immunofluorescence showed the levels of γ-H2AX in U87MG cells stably expressing miR-NC, miR-26a or miR-26a-sponge after TMZ treatments (250 µM, 72 h). γ-H2AX: Green; DAPI: Blue. Scale bar=100 µm. * or # indicated significant difference at *p*<0.05 compared with miR-NC group.

**Supplementary Figure 4 Bax and Bad were decreased in a time-dependent manner under hypoxic condition. (a, b)** U87MG cells were exposed to hypoxia for 0, 12, 24 and 48h, and the expression levels of Bax and Bad were determined by western blotting analysis and qRT-PCR. Data were presented by means ± SEM. in triple experiments. *, ** and *** respectively indicated significant difference at *P*<0.05, *P*<0.01 and *P*<0.001 compared with 0 h group.

**Supplementary Figure 5 MiR-26a/Bax/Bad axis contributes to hypoxia-mediated TMZ resistance *in vitro.*** **(a)** Bax and Bad protein levels were examined by Western blotting to confirm the transfection efficiency. **(b)** Colony formation ability of U87MG cells was measured under normoxia or hypoxia with indicated treatment. **(c)** Staining of Mito-tracker to mark mitochondria primarily, cytochrome c staining to detect the location of cyto-c in U87MG cells co-transfected indicated miRNA mimics and Bax/Bad overexpression or interference after TMZ treatments (250μM) under normoxia or hypoxia. Scale bar=100 µm. Mito-tracker: Red; cytochrome C: Green; DAPI: Blue. **(d)** EdU assay was conducted to detect U87MG cells proliferation upon TMZ treatments (250μM). Scale bar=100 µm. Data were presented by means ± SEM. in triple experiments. * indicates significant difference at *P*<0.01 compared with miR-NC; # and & indicates significant difference compared to miR-26a or miR-26a-inhibitor treatments, respectively.
