# Supplementary material for: Hypoxia-mediated mitochondria apoptosis inhibition induces temozolomide treatment resistance through miR-26a/Bad/Bax axis
Source: Cell Death Dis. 2018 Nov 13;9(11):1128. doi: 10.1038/s41419-018-1176-7 (PMC6233226; doi:10.1038/s41419-018-1176-7)
Supplement: Supplementary file 2 — supplemental-table1 [file 41419_2018_1176_MOESM2_ESM.docx]

**Supplementary Table 2. Primers Used in This Study.**

| **Primer name** | | **Sequence 5'-3'** |
| --- | --- | --- |
| miR-26a RT | CTCAACTGGTGTCGTGGAGTCGGCAATTCAGTTGAGAGCCTATC | |
| miR-26a-F | ACACTCCAGCTGGGTTCAAGTAATCCAGGA | |
| miR-26a-R | TGGTGTCGTGGAGTCG | |
| U6RT | AACGCTTCACGAATTTGCGT | |
| U6-F | CTCGCTTCGGCAGCACA | |
| U6-R | TGGTGTCGTGGAGTCG | |
| WT-Bax-F | CGGGGTACC CTGGACATTGGACTTCCTCCG | |
| WT-Bax-R | CCGCTCGGGCAGAAGGCACTAATCAAGT | |
| MT-Bax-F | CGGGGTACC CTGGACATTGGACTTCCTCCG | |
| MT-Bax-R | CCGCTCGGGCAGAAGGCACTAATGTACG | |
| WT-Bad-F | CGGGGTACC CCCACTGCCCTGGGCAGCCATCTTGAA | |
| WT-Bad-R | CCGCTCGAACATTTGGTAGTGAGCACGGC | |
| MT-Bad-F | CGGGGTACC CCCACTGCCCTGGGCAGCCATCAACAA | |
| MT-Bad-R | CCGCTCGAACATTTGGTAGTGAGCACGGC | |
| Bax-F | CCCGAGAGGTCTTTTTCCGAG | |
| Bax-R | CCAGCCCATGATGGTTCTGAT | |
| Bad-F | CCCAGAGTTTGAGCCGAGTG | |
| Bad-R | CCCATCCCTTCGTCGTCCT | |
| CTDSPL-F | TGCTGAGGGAGGGGAGTGAG | |
| CTDSPL-R | GCAGCATGCCACAGGTTGTC | |
| CTDSPL2-F | ATGTTGGCCAGTCAAGTTCC | |
| CTDSPL2-R | CTGTCACCTCTGGGAGCAG | |
| Promoter-miR-26a-1-F | CGGGGTACCATCATCTTGGCTTGTGTTAC | |
| Promoter-miR-26a-1-R | CCGCTCGAGCTCCTGTGGCTTCATTCC | |
| Promoter-miR-26a-2-F | CCGCTCGAGAGGGCTTGGGTTCTGTCAGTTG | |
| Promoter- miR-26a-2-R | CGGGGTACCGGCAGTCAGAGAAGGCTTCACA | |
| ChIP  miR-26a-1-F | CTGGCTGTGCTGTGATAT | |
| ChIP  miR-26a-1-R | CTCCTGTGGCTTCATTCC | |
| ChIP  miR-26a-2-F | GTAACAGGCAGGGAAATCA | |
| ChIP  miR-26a-2-R | GCTTCACAGAGGAGGAGA | |
