# Supplementary material for: Hypoxia-mediated mitochondria apoptosis inhibition induces temozolomide treatment resistance through miR-26a/Bad/Bax axis
Source: Cell Death Dis. 2018 Nov 13;9(11):1128. doi: 10.1038/s41419-018-1176-7 (PMC6233226; doi:10.1038/s41419-018-1176-7)
Supplement: Supplementary file 3 — supplemental-table2 [file 41419_2018_1176_MOESM3_ESM.docx]

**Supplementary Table 1. Primers Used in This Study.**

| Protein | Antibody company |
| --- | --- |
| Hif-1α | **Cell Signaling Technology( CST)** |
| Caspase3 | **Cell Signaling Technology( CST)** |
| Cleaved-Caspase3 | **Cell Signaling Technology( CST)** |
| Ɣ-H2AX | **Cell Signaling Technology( CST)** |
| Bad | **Cell Signaling Technology( CST)** |
| Bax | **Santa Cruz Biotechnology** |
| PARP | **Cell Signaling Technology( CST)** |
| Cleaved-PARP | **Cell Signaling Technology( CST)** |
| Cytochrome C | **Bioworld** |
| β-actin | **Bioworld** |
| VDAC | **Bioworld** |
| IgG | **Santa Cruz Biotechnology** |
