# Supplementary figures and images for: Hypoxia-mediated mitochondria apoptosis inhibition induces temozolomide treatment resistance through miR-26a/Bad/Bax axis
Source: Cell Death Dis. 2018 Nov 13;9(11):1128. doi: 10.1038/s41419-018-1176-7 (PMC6233226; doi:10.1038/s41419-018-1176-7)

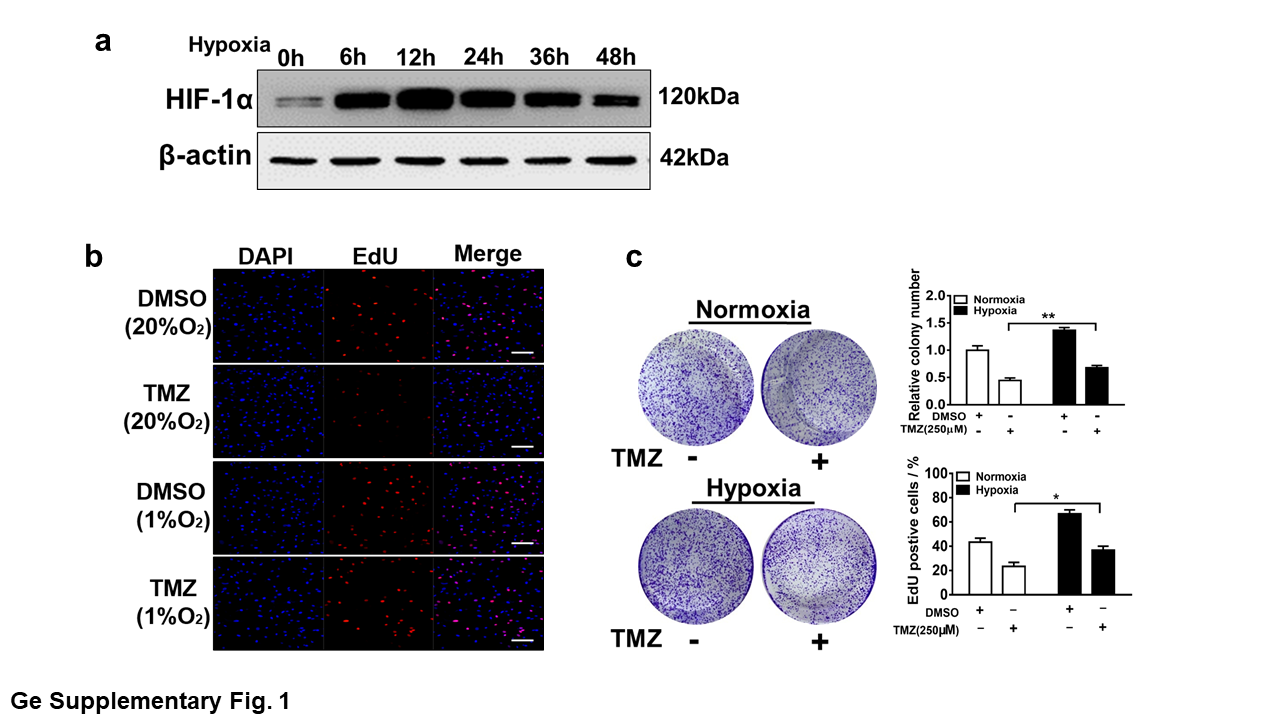

Supplement: Supplementary file 4 — Supplement_Fig1 [file 41419_2018_1176_MOESM4_ESM.tif]

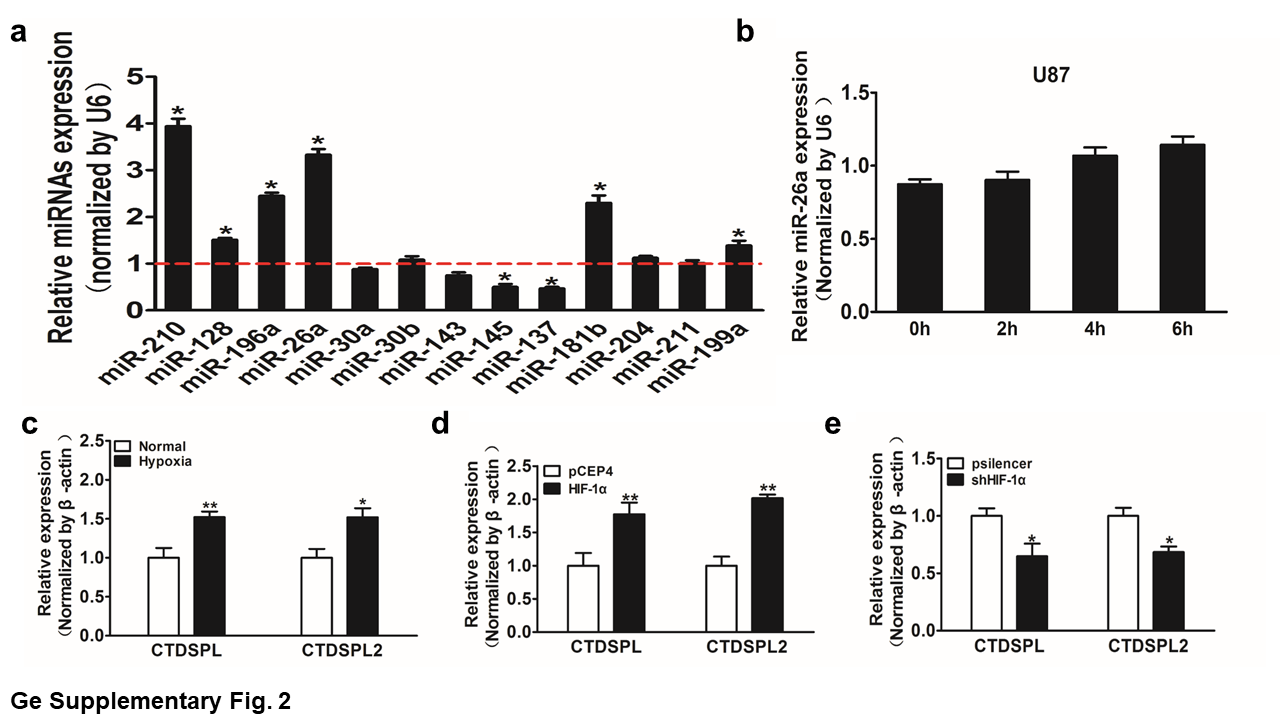

Supplement: Supplementary file 5 — Supplement_Fig2 [file 41419_2018_1176_MOESM5_ESM.tif]

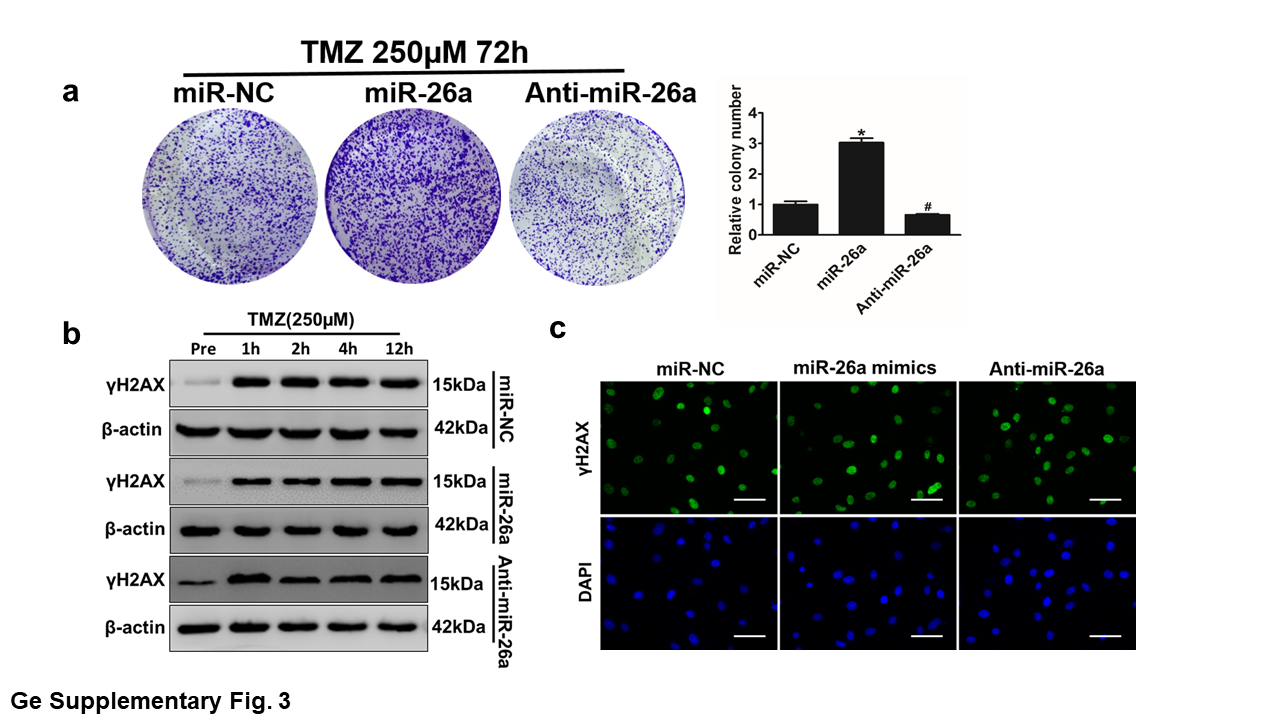

Supplement: Supplementary file 6 — Supplement_Fig3 [file 41419_2018_1176_MOESM6_ESM.tif]

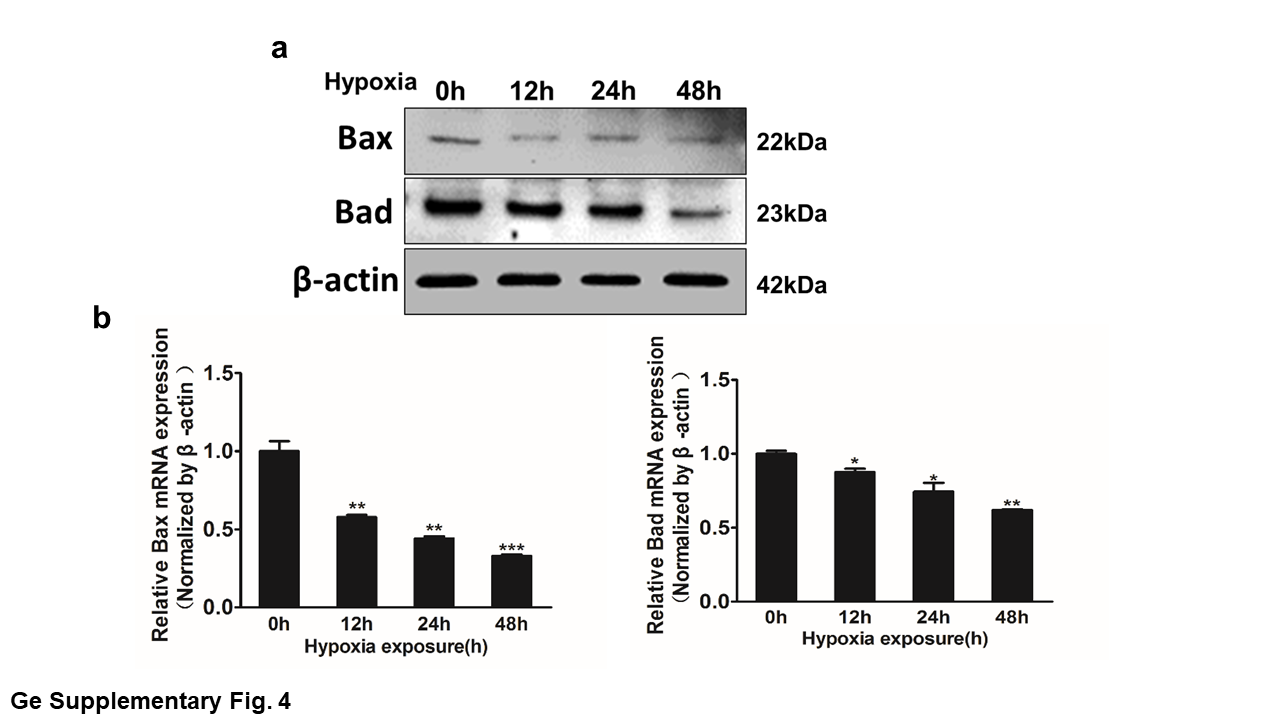

Supplement: Supplementary file 7 — Supplement_Fig4 [file 41419_2018_1176_MOESM7_ESM.tif]

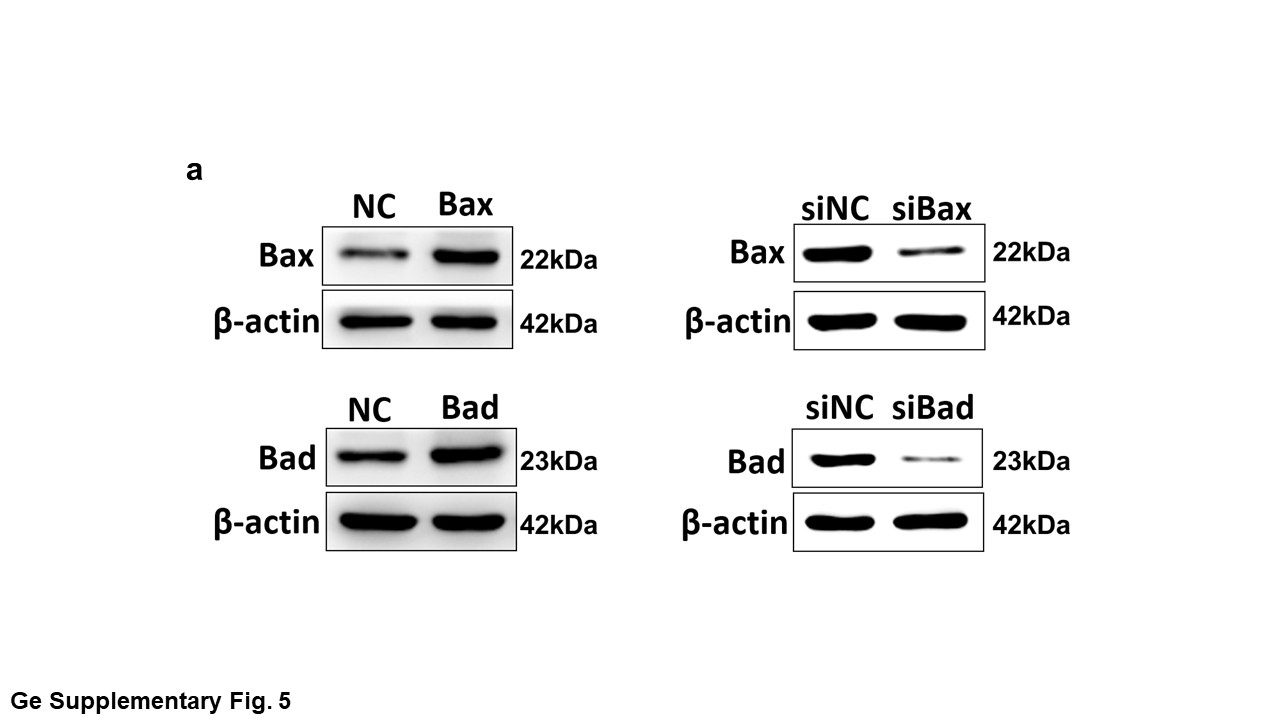

Supplement: Supplementary file 8 — Supplement_Fig5-1 [file 41419_2018_1176_MOESM8_ESM.tif]

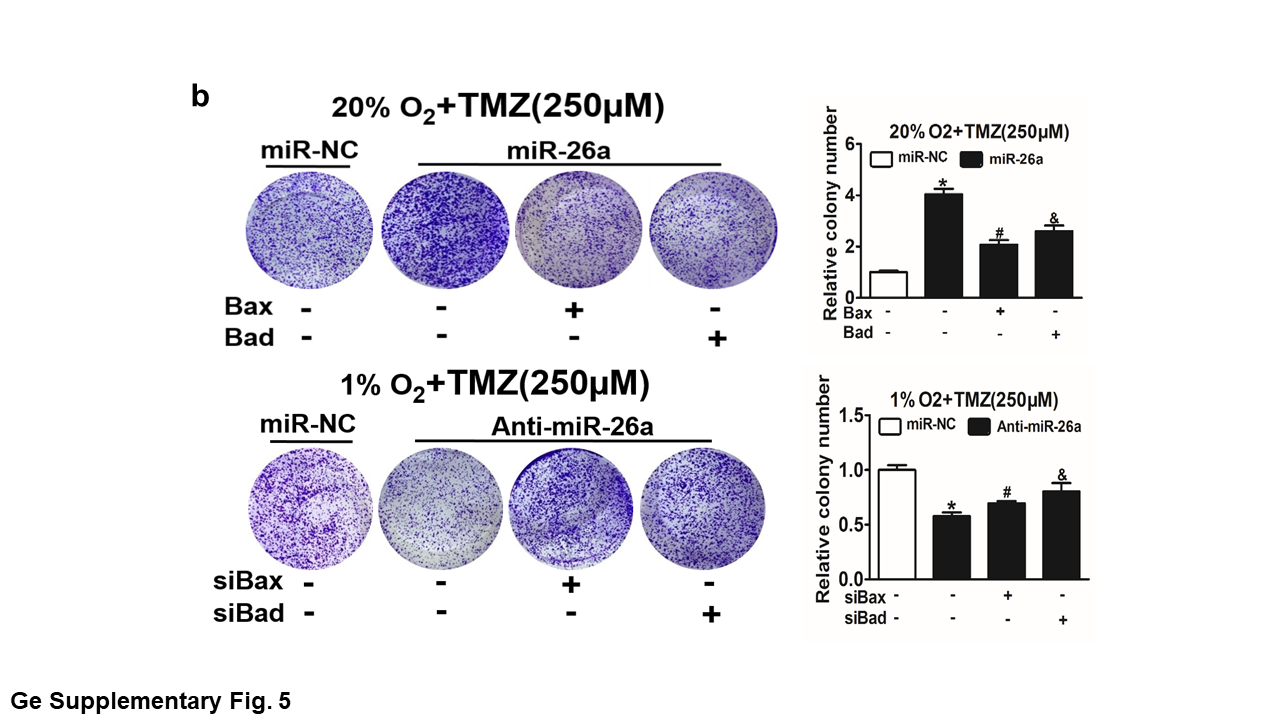

Supplement: Supplementary file 9 — Supplement_Fig5-2 [file 41419_2018_1176_MOESM9_ESM.tif]

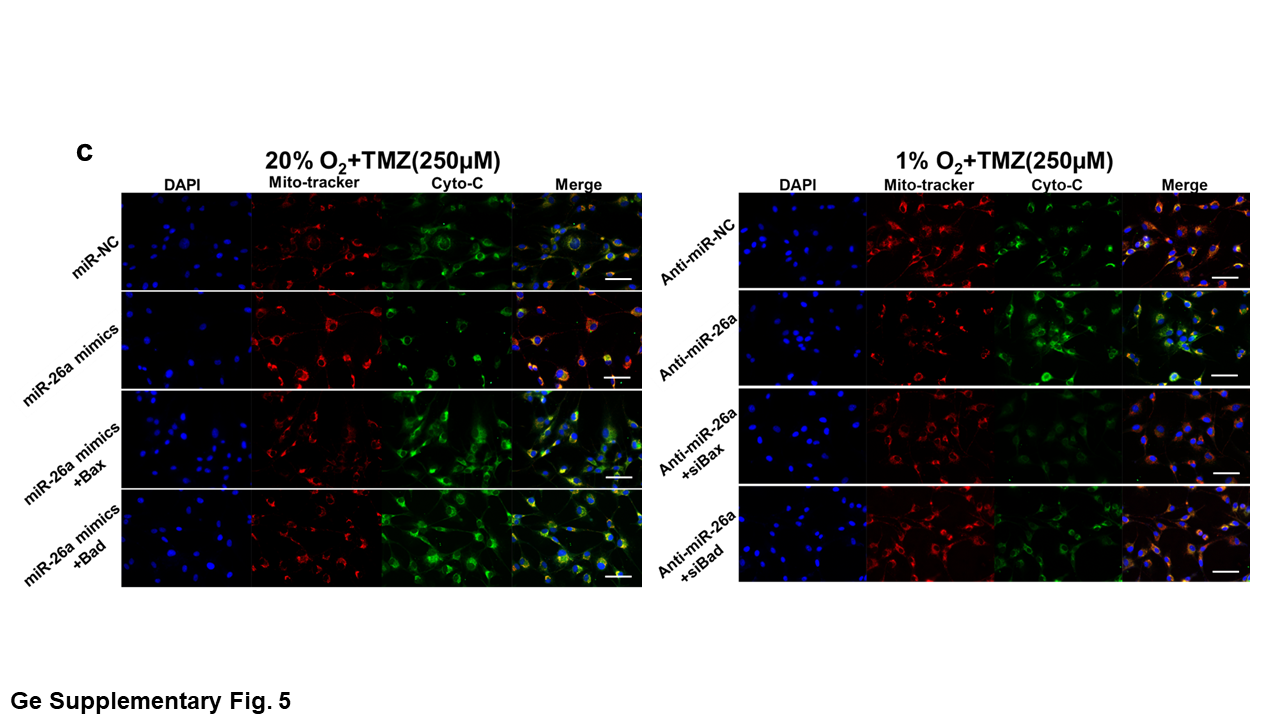

Supplement: Supplementary file 10 — Supplement_Fig5-3 [file 41419_2018_1176_MOESM10_ESM.tif]

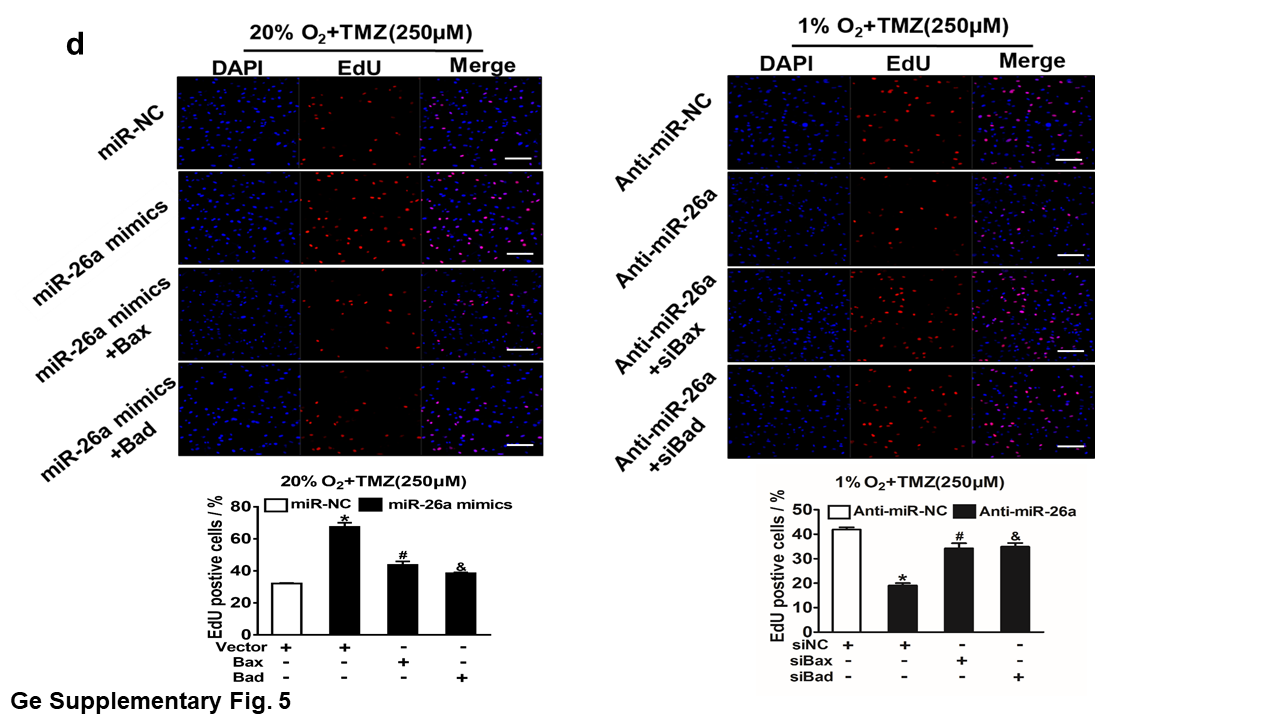

Supplement: Supplementary file 11 — Supplement_Fig5-4 [file 41419_2018_1176_MOESM11_ESM.tif]
